# Supplementary material for: Phosphorylation, Mg-ADP, and Inhibitors Differentially Shape the Conformational Dynamics of the A-Loop of Aurora-A
Source: Biomolecules. 2021 Apr 12;11(4):567. doi: 10.3390/biom11040567 (PMC8070005; doi:10.3390/biom11040567)
Supplement: Supplementary file 1 [file biomolecules-11-00567-s001.pdf]

Supplemental Information

Phosphorylation, Mg-ADP, and Inhibitors Differentially Shape the Conformational Dynamics of the A-loop of Aurora-A

Zahra Musavizadeh [1,*], Alessandro Grottesi [2], Giulia Guarguaglini [3] and Alessandro Paiardini [1,*]

[1] Department of Biochemical Sciences "A. Rossi Fanelli", Sapienza University of Rome, 00185 Rome, Italy
[2] Super Computing Applications and Innovation (CINECA), 00185 Rome, Italy; alegrot@gmail.com
[3] Istituto di Biologia e Patologia Molecolari, Consiglio Nazionale delle Ricerche, 00185 Rome, Italy; giulia.guarguaglini@uniroma1.it
* Correspondence: zahra.musavizadeh@uniroma1.it (Z.M.); alessandro.paiardini@uniroma1.it (A.P.)

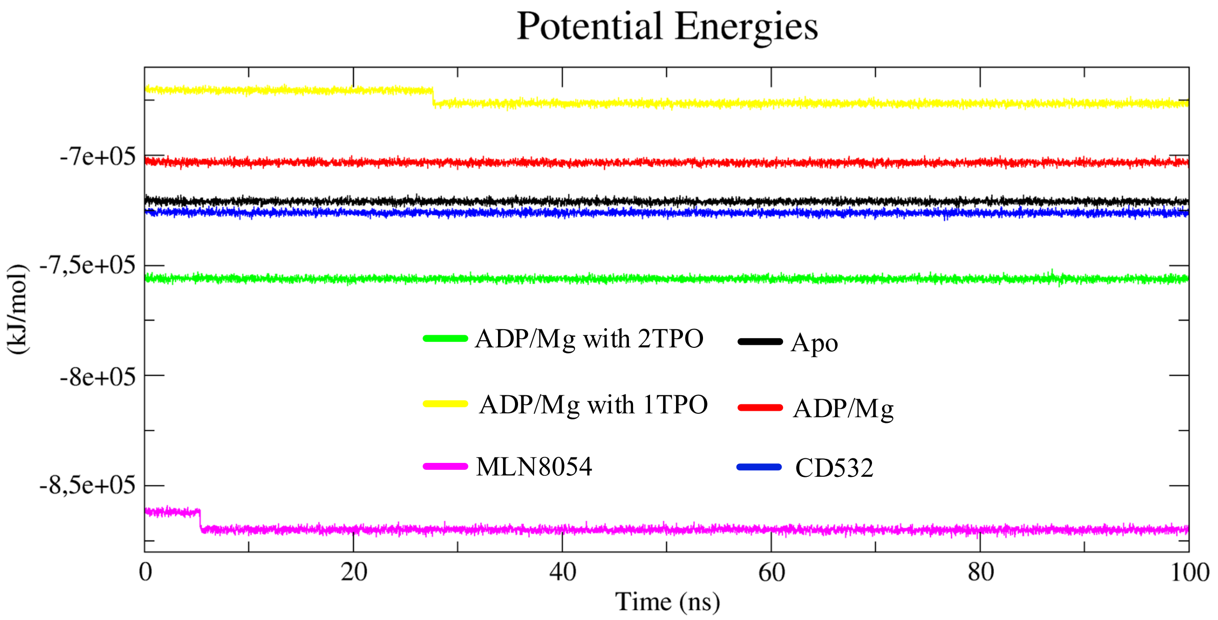

Figure S1. The potential energy of the system. These graphs were calculated during 100 ns MD simulations for each complex. The active structures: Apo (4J8N, black), ADP/Mg (1MQ4, red), ADP/Mg with 2TPO (1OL7, green), ADP/Mg with TPO (1OL7, yellow) and the inactive states: CD532 (4J8M, blue), MLN8054 (2WTV, pink).

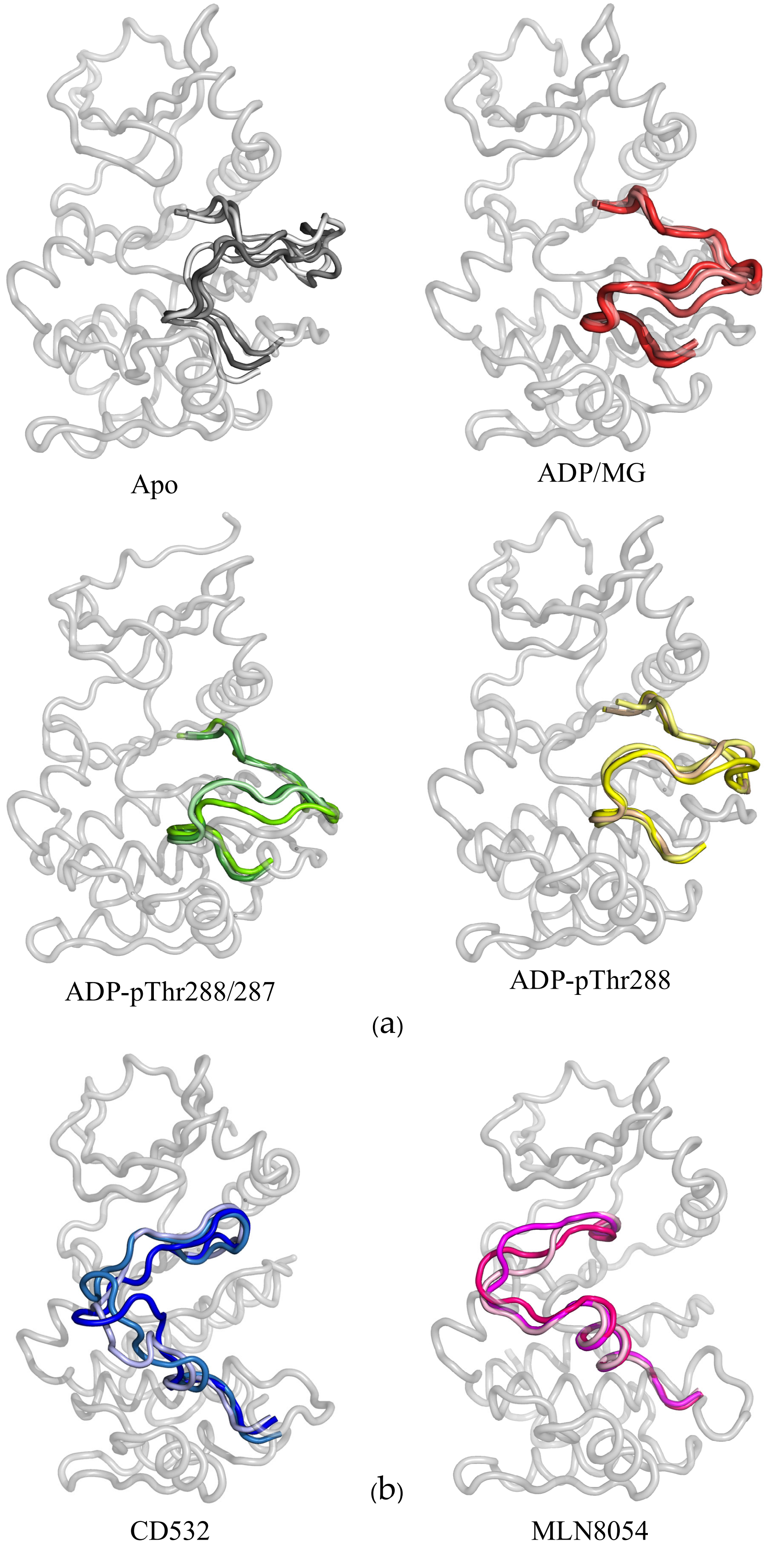

Apo
ADP/MG
ADP-pThr288/287
ADP-pThr288
(a)
(b)
CD532
MLN8054

Protein state	Nr. of Cluster	Cluster 1#	Trans.#1	Cluster 2#	Trans.#2	Cluster 3#	Trans.#3
APO	55	1024	234	868	220	584	54
ADP-MG	23	2852	954	706	472	453	456
ADP/MG-2TPO	20	1726	575	999	309	809	238
ADP/MG-1TPO	12	2137	269	2064	163	94	142
CD532	248	434	177	276	122	193	92
MLN8054	53	1287	370	984	294	521	245

(C)

Figure S2. Representative examples (corresponding to the time frame of the centroid of the first three clusters) showing the activation loop of the Aurora-A protein for 10-100 ns molecular dynamics simulation. (**a**) the active structures: Apo (4J8N, black), ADP/Mg (1MQ4, red), ADP/Mg with 2TPO (1OL7, green), ADP/Mg with TPO (1OL7, yellow). (**b**) the inactive states: CD532 (4J8M, blue), MLN8054 (2WTV, pink).
(**c**) The table reports the total number of clusters obtained in the cluster analysis (see Methods) and the number of members for the first three clusters in each system. The number of cluster members transitions for each of the first three clusters is also reported.

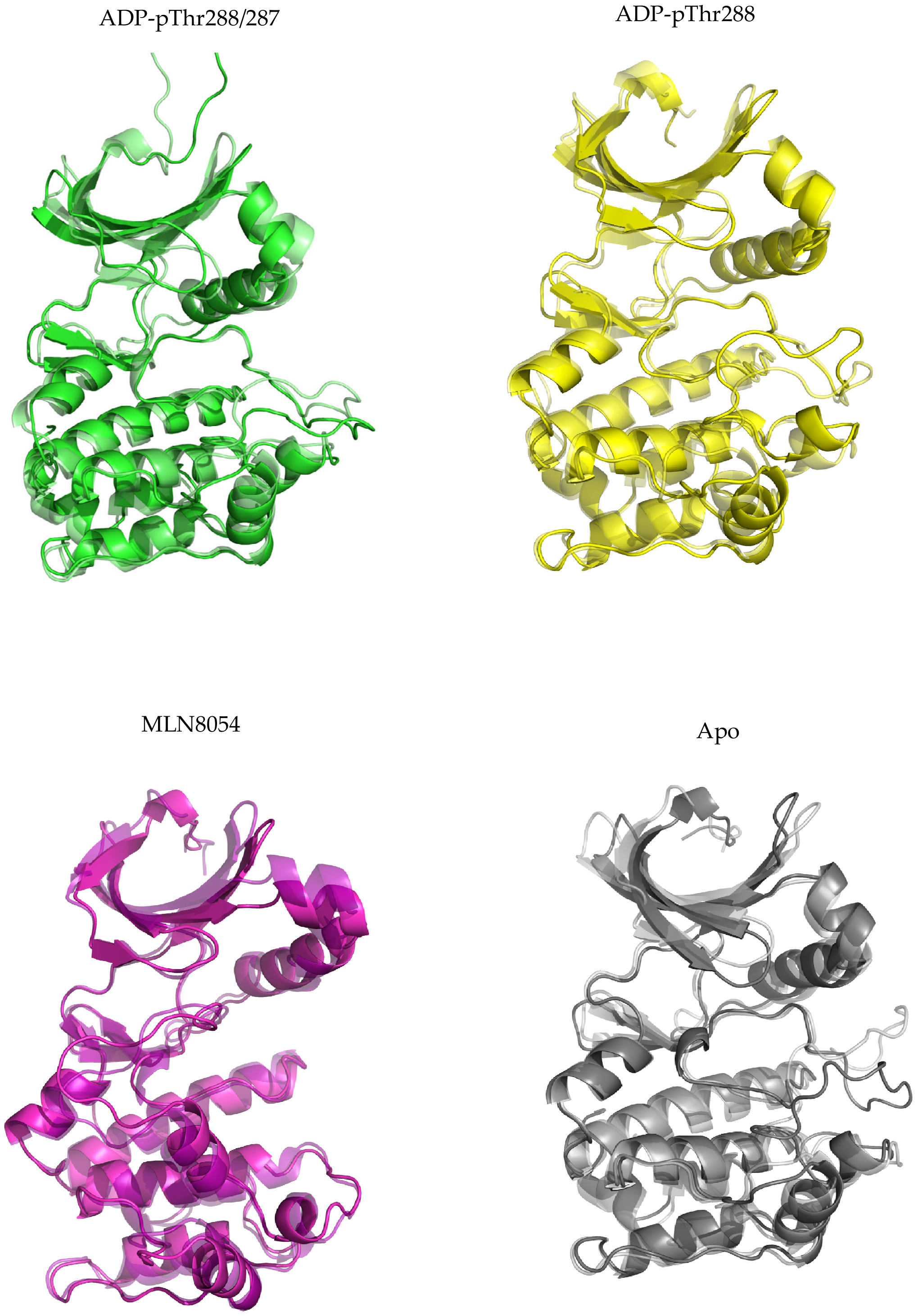

Figure S3. Representative structures of the principal components analysis of the 10-100 ns molecular dynamics simulations of Aurora-A. (a) the active structures: ADP/Mg with 2TPO (1OL7, green), ADP/Mg with TPO (1OL7, yellow) Apo (4J8N, black) and the inactive state: MLN8054 (2WTV, pink).

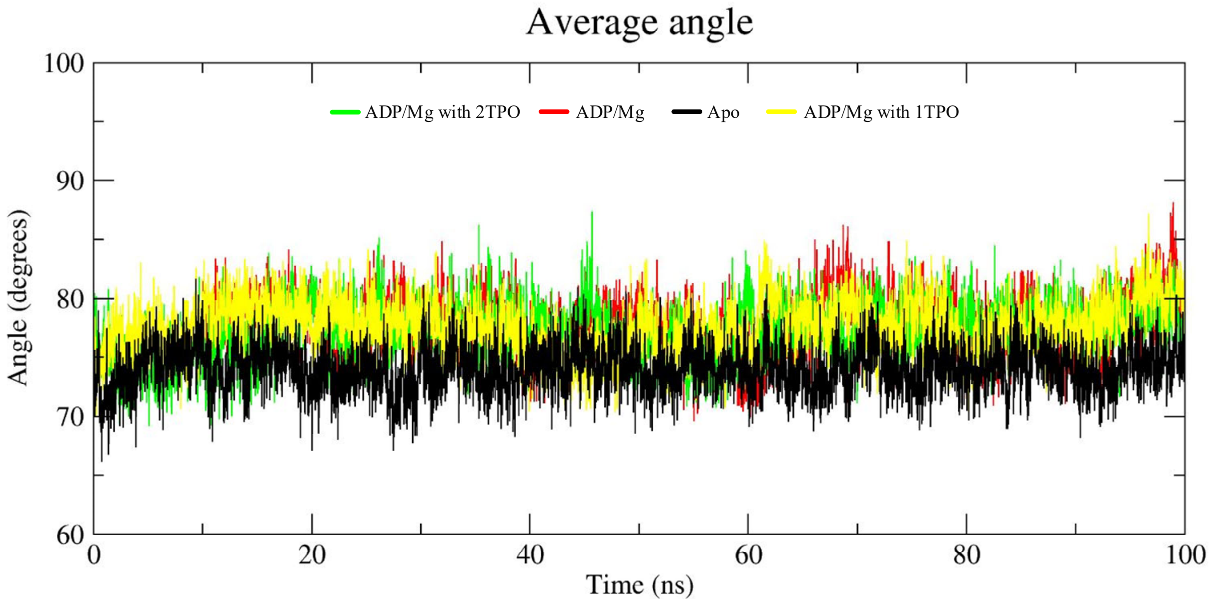

(a)

**Protein state**	Mean	Stdev
CD532	80.9	2.7
MLN8054	79.1	2.1
APO	74.0	2.0
ADP-MG	77.7	2.4
ADP/MG-2TPO	77.1	2.3
ADP/MG-1TPO	77.5	2.3

(b)

Figure S4. (**a**) the plot represents the angle value during the trajectories of Aurora-A for 100 ns MD simulations in the active states: Apo (4J8N, black), ADP/Mg (1MQ4, red), ADP/Mg with 2TPO (1OL7, green), ADP/Mg TPO (1OL7, yellow). (**b**) the table represents the mean angle value between N- and C-lobes during the trajectories of Aurora-A calculated for the 10-100 ns trajectories of MD simulations.
